# Supplementary material for: Incidents of aggression in German psychiatric hospitals: Is there an increase?
Source: PLoS One. 2021 Jan 5;16(1):e0245090. doi: 10.1371/journal.pone.0245090 (PMC7785246; doi:10.1371/journal.pone.0245090)
Supplement: Attachment — Submitted filename: Review_PlosOne.docx [file pone.0245090.s001.docx]

The current study is an interesting multi-center study aimed to investigate whether there is an increase of violent incidents in German Psychiatric Hospitals. This is a very relevant topic, both for staff as well as for patients. In general, the text will benefit from some relatively minor language adjustments. Furthermore, the authors should emphasize on which form of aggression they included and how this form relates to previous literature.

Abstract

1. Was the SOAS-R introduced by the researchers, or by the board of each hospital as a part of care?
2. What is the rationale for the focus on aggressive behaviour towards others? The SOAS-R measures verbal aggression, physical aggression (towards others and towards objects), and aggression against oneself. You should emphasize on this in the manuscript.

Introduction

1. The authors only mention the negative impact on health care workers. However, there is also a burden for patients and their environment, making aggressive behaviour an even more relevant topic to study.
2. Of what kind are the hospitals mentioned in the introduction? Acute, long-term, forensic?
3. The introduction states “We set out to implement a valid and reliable system of data recording in a hospital group”. Although this seems a prospective study, it is mentioned later that the current study is retrospective. This is not clear from the introduction.
4. How does the second research question relate to previous studies?

Methods

1. There are several typos in the manuscript, especially in the methods (goog = good, p.6; independet = independent, p.7; assess = assesses, p.7; behavioud = behaviour, p.9; i = in, p.9).
2. What was the length of stay for forensic psychiatry?
3. How can the maximum score be 21, considering the SOAS-R has 5 aspects with a range from 0 to 4? As described in the article of Nijman et al (1999), the SOAS-R has a range from 0 to 22 and does not use a Likert scale.
4. “However, this changed when staff councils realized that statistics generated from the SOAS files documented the difficulties and dangerousness of their work.” How do you know the staff became aware? Focus groups? Otherwise, you could use a reference to state that implementing something new takes time.
5. Why exclude cases with only self-directed aggression? And how does this relate to previous estimates? Did you also exclude self-directed aggression?

Results

1. Perhaps you could show your main findings in a bar chart?
2. Did you exclude verbal aggression against others as well?
3. Why did you exclude forensic psychiatry from Table 3?

Discussion

1. I would suggest making a conclusion paragraph to end the discussion.
