# Supplementary material for: Incidents of aggression in German psychiatric hospitals: Is there an increase?
Source: PLoS One. 2021 Jan 5;16(1):e0245090. doi: 10.1371/journal.pone.0245090 (PMC7785246; doi:10.1371/journal.pone.0245090)
Supplement: Attachment — Submitted filename: Response letter to Reviewers PloS one.docx [file pone.0245090.s002.docx]

Response letter to Reviewers

We thank the reviewers and the editor as well for their thoughtful and important comments. Thank you for helping us to further improve the manuscript. We followed the points raised by the editor and the reviewers as follows:

*a) If there are ethical or legal restrictions on sharing a de-identified data set, please explain them in detail (e.g., data contain potentially identifying or sensitive patient information) and who has imposed them (e.g., an ethics committee). Please also provide contact information for a data access committee, ethics committee, or other institutional body to which data requests may be sent.*

*b) If there are no restrictions, please upload the minimal anonymized data set necessary to replicate your study findings as either Supporting Information files or to a stable, public repository and provide us with the relevant URLs, DOIs, or accession numbers. Please see* [*http://www.bmj.com/content/340/bmj.c181.long*](http://www.bmj.com/content/340/bmj.c181.long) *for guidelines on how to de-identify and prepare clinical data for publication. For a list of acceptable repositories, please see* [*http://journals.plos.org/plosone/s/data-availability#loc-recommended-repositories*](http://journals.plos.org/plosone/s/data-availability#loc-recommended-repositories)*.*

We uploaded an anonymized data set in Dryad (doi:10.5061/dryad.xpnvx0kdq). We did not know whether this information has to be included into the manuscript directly, in the current version it is not included.

*3. PLOS requires an ORCID iD for the corresponding author in Editorial Manager on papers submitted after December 6th, 2016. Please ensure that you have an ORCID iD and that it is validated in Editorial Manager. To do this, go to ‘Update my Information’ (in the upper left-hand corner of the main menu), and click on the Fetch/Validate link next to the ORCID field. This will take you to the ORCID site and allow you to create a new iD or authenticate a pre-existing iD in Editorial Manager. Please see the following video for instructions on linking an ORCID iD to your Editorial Manager account:* [*https://www.youtube.com/watch?v=_xcclfuvtxQ*](https://www.youtube.com/watch?v=_xcclfuvtxQ)

I did so (Tilman Steinert)

*Reviewers' comments:*

***Comments to the Author***

*3. Have the authors made all data underlying the findings in their manuscript fully available?

The* [*PLOS Data policy*](http://www.plosone.org/static/policies.action#sharing) *requires authors to make all data underlying the findings described in their manuscript fully available without restriction, with rare exception (please refer to the Data Availability Statement in the manuscript PDF file). The data should be provided as part of the manuscript or its supporting information, or deposited to a public repository. For example, in addition to summary statistics, the data points behind means, medians and variance measures should be available. If there are restrictions on publicly sharing data—e.g. participant privacy or use of data from a third party—those must be specified.*

Reviewer #1: Yes

Reviewer #2: No

See above, we provided the data in a publicly accessible repository that will be activated if the article is published

4. Is the manuscript presented in an intelligible fashion and written in standard English?

PLOS ONE does not copyedit accepted manuscripts, so the language in submitted articles must be clear, correct, and unambiguous. Any typographical or grammatical errors should be corrected at revision, so please note any specific errors here.

Reviewer #1: Yes

Reviewer #2: No

We sent the manuscript for proofreading and corrected grammar errors and typos. Please note that we changed the title from “aggressive incidents…“ into “incidents of aggression“ according the proof reader’s suggestion

*Reviewer #1: The manuscript clearly describes the different sections of methodology, results and conclusions, responding to the proposed objectives. It is written clearly and simply, easy to read. There are 3 things I would like to review:

In the first place it would be good to comment in the text that there is evidence of a generalized increase in aggressions in society in general and especially in the workplace, but especially in the health sector. In addition, there are studies that show that this violence is growing in all health areas, not only in psychiatry, there are even authors who assure that there is a higher rate of aggression in other units, such as in primary care. I think it would be good to mention this and then talk specifically about assaults in psychiatry.*

These issues are complex. Violence against those groups are an issue of increasing public concern, but good evidence from longitudinal studies is missing similarly like in our topic.

We added the following sentences and included further references: Violent crime has been more or less continuously decreasing in the Western world for decades [4,5]; however, violence against paramedics, figherfighters, and medical staff has been receiving increasing attention, is designated as “unacceptable” by the World Health Organization [6], and is a frequent phenomenon across countries [7]. It is unclear whether this is due to a real increase or rather to an increased propensity to report such incidents. Long-term, large-scale studies using identical instruments for measurement are lacking. *Secondly, in the discussion, I believe that the point where the possible bias is discussed when comparing the results with those of other countries should be developed a little more, since psychiatry is not so developed in all countries nor are there hospitals so specific to treat these patients, these patients are sometimes in general hospital, this can make it difficult to compare with data from other countries.*

Both in the introduction and in the discussion section we had mentioned that the available meta-analysis by Iozzino et al. referred to facilities in high-income countries. We added the following sentence: „With respect to countries with low and middle income where in-patient psychiatric treatment frequently takes place in general hospitals and not in separated psychiatric facilities, we cannot draw any conclusions from the data.” *And thirdly, I believe that it should also be considered that despite passing a scale to assess the aggressions received, it should be taken into account that it is a very subjective issue, since not all people perceive violence in the same way, there is Studies that show that there are health workers who take aggressions as an inherent part of their work and even more so if they are personnel who work with psychiatric patients.*

We added the following sentences:

Reasons for improved reporting might be not only of administrative character. Even if we had introduced a validated instrument by using the SOAS-R, the subjective perception of violence and, associated with it, the threshold to fill a form, might have changed within the observed years due to increased public awareness. While in former years, staff in psychiatric facilities frequently viewed at least milder forms of violence by inpatients as “part of the job,” nowadays, each kind of violence is considered unacceptable [6]; rather, an attitude of “zero tolerance” prevails.

”.

*Reviewer #2: The current study is an interesting multi-center study aimed to investigate whether there is an increase of violent incidents in German Psychiatric Hospitals. This is a very relevant topic, both for staff as well as for patients. In general, the text will benefit from some relatively minor language adjustments. Furthermore, the authors should emphasize on which form of aggression they included and how this form relates to previous literature.*

We sent the text to a proofreading service.

*Abstract
1. Was the SOAS-R introduced by the researchers, or by the board of each hospital as a part of care?
2. What is the rationale for the focus on aggressive behaviour towards others? The SOAS-R measures verbal aggression, physical aggression (towards others and towards objects), and aggression against oneself. You should emphasize on this in the manuscript.*

We clarified that SOAS-R was introduced as part of routine documentation and why we did not analyse self-directed aggression. The method section was changed as follows: :In a group of 10 hospitals serving about half of the population of the Federal State of Baden-Wuerttemberg with 11 million inhabitants, the Staff observation aggression Scale, revised (SOAS-R) was introduced into the electronic charts as part of routine documentation. Data recording was strongly supported by staff councils and unions. Complete data is now available for the year 2019. For one hospital, data is available since 2006. Due to some doubts with respect to fully covering self-directed aggression, we restricted the analysis to aggression toward others and toward objects.

*Introduction
1. The authors only mention the negative impact on health care workers. However, there is also a burden for patients and their environment, making aggressive behaviour an even more relevant topic to study.*

We added the following sentence and a reference: Similarly, fellow patients may feel intimidated and suffer from an environment perceived as unsafe and potentially traumatizing (8).

*2. Of what kind are the hospitals mentioned in the introduction? Acute, long-term, forensic?*

A distinction between acute and long-term hospitals does not exist in Germany. We had mentioned that the 10 hospitals serve half of the population with defined catchment areas and that the mean length of stay is 25.3 days (section “setting“). With respect to forensic hospitals, we had written … ”provide the complete amount of the State’s forensic hospital beds”. Hence to our mind it should be sufficiently clear that the hospitals provide all types of in-patient mental health care for a considerable part of the population. Therefore we had written ”… the sample can be considered as approximately representative for the Federal State”. We have no good ideas about helpful further clarifications.

*3. The introduction states “We set out to implement a valid and reliable system of data recording in a hospital group”. Although this seems a prospective study, it is mentioned later that the current study is retrospective. This is not clear from the introduction.*

We think that this is sufficiently clarified in the methods section. In all studies on incidents of any kind, data recording can be planned prospectively, but the incidence of the respective incidents is then analysed retrospectively for a defined period of time, just like counting adverse effects in prospectively planned phase IV studies of drugs.

*4. How does the second research question relate to previous studies?*

The second research question (comparing general psychiatry, adolescent psychiatry, and forensic psychiatry) is of explorative nature. We are not aware of any previous studies that had did that kind of analysis, but we did not want to expand this section with a discussion of not existing literature.  *Methods
1. There are several typos in the manuscript, especially in the methods (goog = good, p.6; independet = independent, p.7; assess = assesses, p.7; behavioud = behaviour, p.9; i = in, p.9).*

Thank you, corrected. We perform always worse in finding our own mistakes than finding those of others…

*2. What was the length of stay for forensic psychiatry?*

In the section “ setting“, we added the following sentence: “In the forensic hospitals, length of stay is about five years, varying between hospitals and across time.” As in forensic psychiatry several patients stay for an undefined time, depending on courts’ decision, an exact determination of average length of stay is possible only many years later.

*3. How can the maximum score be 21, considering the SOAS-R has 5 aspects with a range from 0 to 4? As described in the article of Nijman et al (1999), the SOAS-R has a range from 0 to 22 and does not use a Likert scale.*

Oh sorry, thank you to recognize this error (22 instead of 21).

*4. “However, this changed when staff councils realized that statistics generated from the SOAS files documented the difficulties and dangerousness of their work.” How do you know the staff became aware? Focus groups? Otherwise, you could use a reference to state that implementing something new takes time*

We added the following sentence: ’Reasons for improved reporting might be not only of administrative character. Even if we had introduced a validated instrument with SOAS-R, the subjective perception of violence and, associated with it, the threshold to fill a form, might have changed within the observed years due to the increased public awareness. While in former years, staff in psychiatric facilities frequently viewed at least milder forms of violence by in-patients as “part of the job”, nowadays each kind of violence is considered as unacceptable (6) and an attitude of “zero tolerance” is prevailing.’

5. *Why exclude cases with only self-directed aggression? And how does this relate to previous estimates? Did you also exclude self-directed aggression?*
We added the following sentence in the implementation section: ’As the SOAS-R is viewed and has been introduced as an instrument to record patients’ aggressive behaviour towards others, particularly staff, we have some doubts whether self-directed aggressive behaviour, which was not objective of this study, is recorded completely. ’ And in the analysis section (2nd sentence): ’We excluded cases with only self-directed aggression (3.4 % of reported cases) due to suspected underreporting. ’

*Results
1. Perhaps you could show your main findings in a bar chart?*

We discussed that. We think that presenting findings as a bar chart would mean a loss of information compared to the tables, and presenting the same findings in a table and a figure as well would not be allowed according to journal style.

*2. Did you exclude verbal aggression against others as well?*

No. We refer to the desciption ’Aggression is defined as any verbal, non-verbal, and/or physical behaviour that is either threatening or actually harmful to persons or property.’ Because verbal aggression was not excluded, we saw no reason to mention that explicitly.

*3. Why did you exclude forensic psychiatry from Table 3?*

Merging results from general hospital psychiatry and forensic psychiatry would be misleading. Instead, to present results from forensic psychiatry, we added another table for results in forensic psychiatry.

*Discussion
1. I would suggest making a conclusion paragraph to end the discussion.*

 We did so: This is the most robust estimate of the frequency of violent incidents in German psychiatric hospitals so far. The incidence is about half of what has been reported internationally, probably due to sample selection bias in previous studies and a relatively high number of hospital beds in Germany. Available data suggests an increase of violent incidents over the last ten years; however, it is unclear to which extent this is due to increased reporting.
